# Novel pathway of cypermethrin biodegradation in a *Bacillus* sp. strain SG2 isolated from cypermethrin-contaminated agriculture field

**DOI:** 10.1007/s13205-016-0372-3

**Published:** 2016-02-04

**Authors:** Pankaj, Anita Sharma, Saurabh Gangola, Priyanka Khati, Govind Kumar, Anjana Srivastava

**Affiliations:** 1 Department of Microbiology, College of Basic Sciences and Humanities, G.B Pant University of Agriculture and Technology, Pantnagar, 263145 India; 2 Department of Chemistry, College of Basic Sciences and Humanities, G.B Pant University of Agriculture and Technology, Pantnagar, 263145 India

**Keywords:** *Bacillus* sp., Biodegradation, Cypermethrin, GC–MS, Pyrethroid

## Abstract

Pesticides belonging to pyrethroid group are widely used in agricultural fields to check pest infestation in different crops for enhanced food production. In spite of beneficial effects, non-judicious use of pesticides imposes harmful effect on human health as their residues reach different food materials and ground water via leaching, percolation and bioaccumulation. Looking into the potential of microbial degradation of toxic compounds under natural environment, a cypermethrin-degrading *Bacillus* sp. was isolated from pesticide-contaminated soil of a rice field of Distt. Udham Singh Nagar, Uttarakhand, India. The bacteria degraded the compound up to 81.6 % within 15 days under standard growth conditions (temperature 32 °C pH 7 and shaking at 116 rpm) in minimal medium. Analysis of intermediate compounds of biodegraded cypermethrin revealed that the bacteria opted a new pathway for cypermethrin degradation. GC–MS analysis of biodegraded cypermethrin showed the presence of 4-propylbenzoate, 4-propylbenzaldehyde, phenol M-tert-butyl and 1-dodecanol, etc. which was not reported earlier in cypermethrin metabolism; hence a novel biodegradation pathway of cypermethrin with *Bacillus* sp. strain SG2 is proposed in this study.

**Electronic supplementary material:**

The online version of this article (doi:10.1007/s13205-016-0372-3) contains supplementary material, which is available to authorized users.

## Introduction

Pyrethroid insecticides are synthetic derivatives of compounds of pyrethrin, produced by *Chrysanthemum* plants (Soderlund et al. 2002). These pesticides are widely used in agriculture, forestry, horticulture, public health and homes as well as for the protection of textiles and buildings (Lin et al. 2011; Zhang et al. 2011). In agriculture, these pesticides are being used for more than 40 years (Narahashi et al. 1998; Shafer et al. 2005). Extensive use of these insecticides not only results in pest resistance but also leads to several environmental issues including human health (Li et al. 2008). Cypermethrin is mainly used in cereal and ornamental plants against coleopteran (*Tribolium confusum*), lepidepteran and other broad group of insect larva. This pesticide is also toxic for fish. Freshwater fish (*Channa punctatus*) exposed to cypermethrin show reduced resistance because of low levels of red blood cells and proteins in the blood (Saxena and Saxena 2010). High levels of this chemical are neurotoxic and cause ataxia, excessive salivation, choreoatheosis, tremors and convulsions in rabbits (Khanna et al. 2002). Cypermethrin affects the voltage-dependent sodium channel and ATPase system in neuronal membranes. It binds to nuclear DNA and leads to destabilization and unwinding of DNA (Patel et al. 2006).

Looking into the facts of toxicity and persistency of this pesticide, it is urgently required to develop some strategies to eliminate or detoxify cypermethrin and its metabolites from the environment. Microbial diversity is the major factor in determining the fate of numerous xenobiotic/recalcitrant compounds in a contaminated environment. Biodegradation process involves the use of living microorganisms to detoxify or degrade the pollutants to less toxic forms. Till date, several bacterial strains such as *Pseudomonas aeruginosa* (Zhang et al. 2011), *Streptomyces sp.* (Lin et al. 2011), *Stenotrophomonas sp.* (Chen et al. 2011a) and *Serratia marcescens* (Cycoń et al. 2014a, b) have been reported to degrade pyrethroid pesticides. Many enzymes involved in biodegradation of cypermethrin and other pyrethroids are reported by Zhai et al. (2012) and Fan et al. (2012).In the present study, a *Bacillus* sp. (SG2) able to degrade cypermethrin was recovered from the pesticide-contaminated soil of a rice field of Tarai region of Uttarakhand. Biodegradation products of cypermethrin were analysed and possible biodegradation pathway of cypermethrin by *Bacillus* sp. (SG2) is proposed.

## Materials and methods

### Chemicals and media

Technical grade cypermethrin (95 % pure) was obtained from Department of Chemistry of the University and dissolved in acetonitrile to make a stock of 1 mg/ml. Stock solution was filter sterilized and kept in refrigerator for use. Minimal salt medium and nutrient broth (pH 7.0) were used for the isolation and cultivation of pesticide-degrading bacterial strains according to Negi et al. (2014). All the chemicals and solvents used in this study were of analytical grade.

### Screening and isolation of cypermethrin-degrading bacteria

Pesticide-contaminated soils were collected from a rice field of Udham Singh Nagar, Uttarakhand, India. As per the result of residual analysis of the pesticide, the soil samples were used for the isolation of cypermethrin-degrading bacteria. One gramme soil was suspended in sterile distilled water. After dilution, one ml of soil suspension (10^−5^) was inoculated in nutrient agar plates. Bacterial colonies that appeared after 24 h on nutrient agar were enriched with cypermethrin by growing them successively in minimal medium. Discrete and pure bacterial colonies were transferred to 50 ml nutrient broth and incubated at 30 °C on an incubator shaker at 100 rpm. Cypermethrin-degrading bacterial cultures were screened from the isolated pure bacterial cultures by growing them in minimum salt agar plates supplemented with 50 ppm cypermethrin as described by Xu et al. (2008) and Negi et al. (2014). Selected bacteria (SG2) utilized cypermethrin as a carbon and energy source. Biodegradation studies of cypermethrin were monitored by analysing residual cypermethrin and its intermediates (after extraction) using a high-performance liquid chromatography system (HPLC–Dionex) at Department of Chemistry.

### Identification of cypermethrin degrading bacterial strain

Microbiological and biochemical characterization of the cypermethrin-degrading bacterial strain (SG2) was performed according to Holt et al. (1994). For molecular characterization, genomic DNA of SG2 was extracted according to Bazzicalupo and Fani (1995). Amplification of 16S rDNA was done using Universal primers, 5′-AGAGTTTGATCCTGGCTCAG-3′ and 5′-TACCTTGTTACGACTT-3′. PCR conditions used for the present study were initial denaturation at 95 °C for 1 min, annealing temperature 55–51 °C (touchdown) followed by 35 cycles of denaturation at 94 °C for 30 s, annealing at 51 °C for 30 s and extension at 72 °C for 1 min, with the last cycle followed by 10 min extension at 72 °C. After gel electrophoresis, PCR product was eluted using an extraction kit (Genei). After quantification, the sample was sent to Advanced Biotechnology Centre (CIF), Delhi University South Campus, India, for sequencing. Obtained sequences were compared with GenBank database using BLAST programme. Multiple sequence alignment for the homologous sequence was done by MEGA 5.0 software, and a phylogenetic tree was constructed using neighbour-joining method (Tamura et al. 2007).

### Growth-linked biodegradation of cypermethrin in SG2

*In vitro* biodegradation of cypermethrin was performed in 100 ml flask with 50 ml minimal salt medium, supplemented with 50 ppm cypermethrin and inoculated with 1 ml SG2 culture before optimization the biodegradation conditions. Bacterial inoculum was prepared by inoculating single colony in 50 ml of MSM. Inoculated flask was incubated in an incubator shaker @ 100 rpm at 30 °C. Bacterial cells from the log phase, harvested by centrifugation (5000 rpm for 10 min) and washed with minimal medium were used for the subsequent studies. Uninoculated medium acted as control. Bacterial growth was monitored by taking absorbance using UV–Visible spectrophotometer (Perkin Elmer) and the residual cypermethrin concentration was measured by HPLC (Dionex), after extraction according to Anastassiades et al. (2003).

### Optimization of culture conditions for cypermethrin biodegradation

Response surface methodology (RSM) was explored to optimize the degradation conditions of cypermethrin using strain SG2. The Box-Behnken design with three replicates at the centre point was used to optimize the independent variables which significantly influenced cypermethrin biodegradation with *Bacillus* sp. according to Xiao et al. (2015). Three critical factors and their optimal ranges selected for biodegradation of cypermethrin in this experiment were temperature (28–38 °C), pH (4–8) and shaking (90–110 rpm) (Table 1). The dependent variable was degradation of 50 ppm cypermethrin in 50 ml of minimal medium at 15th day.

**Table 1:** Box-Behnken design and the response of dependent variable in cypermethrin biodegradation

Run	X1	X2	X3	Percent degradation by SG2
1	28	5	90	71.4
2	36	5	90	65.6
3	28	9	90	77
4	36	9	90	74.5
5	28	5	110	65.7
6	36	5	110	67.9
7	28	9	110	74
8	36	9	110	69.7
9	25.2	7	100	69.8
10	38.7	7	100	68.9
11	32	3.6	100	64.8
12	32	10.3	100	61.2
13	32	7	83.1	76.5
14	32	7	116.8	81.6
15	32	7	100	80
16	32	7	100	74.7
17	32	7	100	74
18	32	7	100	73
19	32	7	100	72
20	32	7	100	77

The data are means of three replicates

*X*1 temperature, (25, 28, 32, 36 and 38 °C); *X*2 medium pH, (3.6, 5, 7, 9, 10); *X*3 Shaking speed in rpm, (83.1, 90, 100, 110, 116.8)

### FT-IR analysis of cypermethrin biodegradation

An in vitro pesticide biodegradation study was conducted in 50 ml minimal salt medium, inoculated with1 ml of 24-h-old bacterial culture and supplemented with cypermethrin (50 ppm). Uninoculated flasks spiked with same concentration of cypermethrin acted as control. Experiment was conducted in triplicate. Intermediate compounds of cypermethrin were extracted in acetonitrile after 15 days according to Negi et al. (2014). Samples were analysed by FT-IR at Department of Biophysics to locate the bond stretching in the test pesticide after biodegradation.

### GC–MS analysis of cypermethrin biodegradation

To check the degradation of cypermethrin in soil slurry, 50 g autoclaved soil was taken in a 100-ml flask, to which 20 ml of minimal medium was added. Cypermethrin (100 ppm) was added to all the sets, to which 1 ml of 24-h-old bacterial culture was added and mixed properly. One ml of soil slurry was taken from all the flasks separately on 0, 10 and 15th day after incubation. Uninoculated flasks spiked with pesticides acted as control. Extraction and quantification of cypermethrin was done by HPLC. For GC–MS analysis, samples were extracted from soil slurry and metabolites of biodegraded cypermethrin were identified at AIRF facility, JNU, New Delhi.

### Chemical analysis

Residual analysis of cypermethrin in collected soil samples was performed according to Anastassiades et al. (2003). Residual pesticide was extracted by adding either 5 ml of culture broth or 5 g of soil to 20 ml of acetone in a flask. The mixture was filtered using Buchner funnel after shaking for 1 h and the obtained residue was filtered again after washing thoroughly with 10 ml acetone. Filtrate was collected in a round-bottom flask. The residual analysis of the pesticide was done using HPLC attached with C18 reverse-phase column and UV detector. A mixture of acetonitrile and water (70:30, v/v) was used as a mobile phase at a flow rate of 1.0 mL min^−1^ with the injection volume of 10 μL.

## Results

### Isolation and characterization of cypermethrin-degrading bacteria

**Fig. 1:**
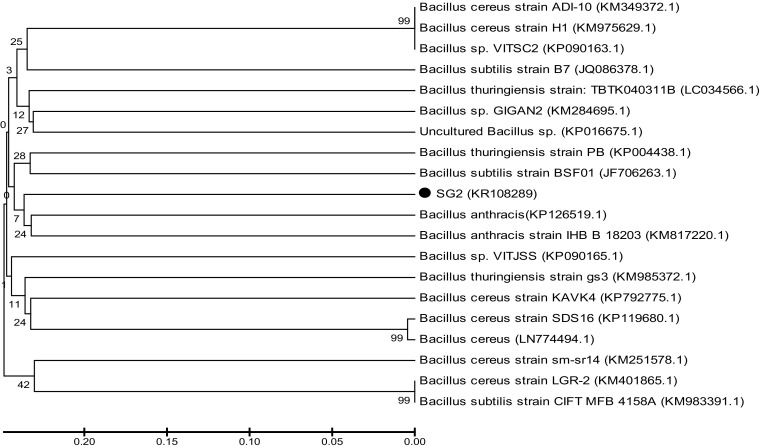
Phylogenetic analysis of SG2 strain and related strains on the basis of 16S rDNA using MEGA 5.0 software. The* numbers* in *parentheses* represent the sequence accession number in Genbank. *Bar* represents sequence divergence

In the present study, a bacterial isolate (SG2), able to utilize cypermethrin as a carbon source, was isolated from the pesticide-contaminated soil of a rice field of Udham Singh Nagar Uttarakhand, India. Bacterial colonies, growing on nutrient agar plates, were rough, opaque and dirty white.The organism was aerobic, gram-positive, rod-shaped and showed positive tests for the production of Indole -3 acetic acid and siderophore and phosphate solubilisation. The bacteria utilized dextrose and mannitol as carbon source and degraded 80 % of cypermethrin (50 ppm) within 15 days under shaking conditions in minimal medium. Analysis of the 16S rDNA gene sequences demonstrated that SG2 belonged to *Bacillus* sp. The organism was provided an accession number (KR108289) by NCBI data base (Fig. 1).

### Utilization of cypermethrin by strain SG2

**Fig. 2:**
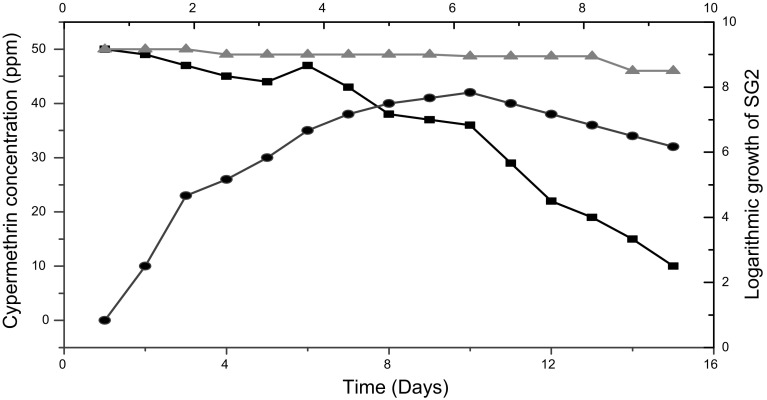
Utilization of cypermethrin by strain SG2 (*filled circle*) growth of SG2, (*filled square*) utilization kinetics of cypermethrin, (*filled triangle*) Control

The isolate, *Bacillus* sp. (SG2), was able to grow on MSM agar plates supplemented with 110–170 ppm cypermethrin. Degradation rate of cypermethrin increased gradually as the strain entered the logarithmic phase (4–5 days) followed by stationary phase (7–8 days) (Fig. 2). A decline in degradation rate was observed when the strain attained the death phase (12 days). More than 81 % of cypermethrin (50 ppm) was degraded by strain SG2 within 15 days in comparison to control.

### Optimization of cypermethrin degradation

**Fig. 3:**
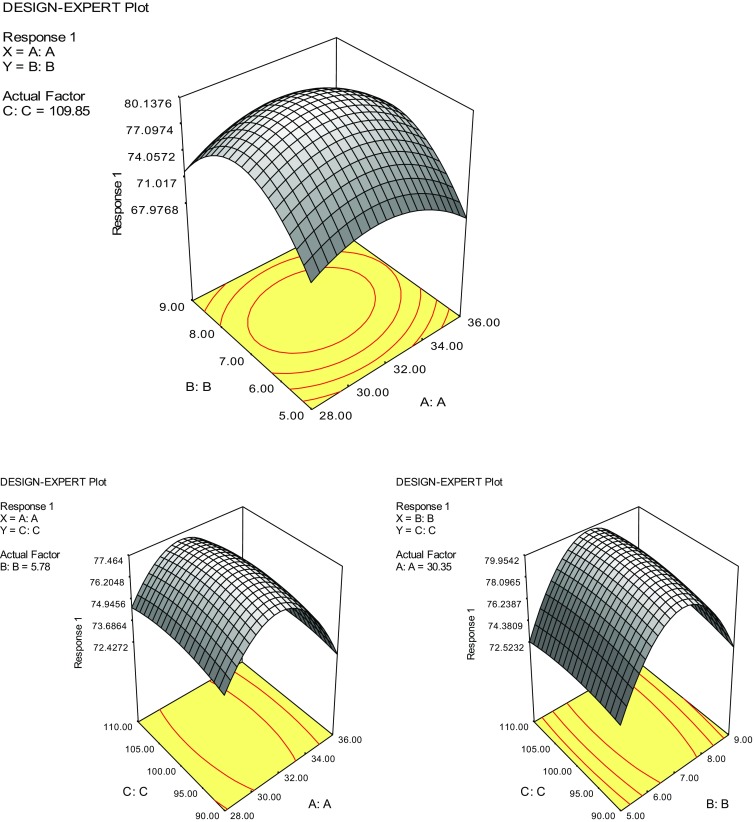
Response surface plot showing effect of temperature, pH and shaking speed on cypermethrin biodegradation (where *A t*emperature, *B* pH, *C* shaking speed)

Box-Behnken design was used to determine the effect of three important variables [temperature (*X*1), pH (*X*2) and shaking speed (*X*3)] on the biodegradation of cypermethrin by *Bacillus* sp. (SG2). The experimental design and the response of dependent variables for cypermethrin are presented in Table 1. Data from Table 1 were processed by response surface regression procedure of Design Expert 6.0.10 software, and results were obtained by fitting the values in the quadratic model equation:

$${\text{Response (}}Y ) = + 80.21 - 0.63X1 + 0.85 \, X2 + 0.32X3 - 3.53 \, X1^{2} - 6.17 \, X2^{2} - 0.49 \, X3^{2} + 0.47X1X2 - 0.10 \, X1X3 + 0.32 \, X2X3$$

where (*Y*) is the predicted cypermethrin degradation (%) by strain SG2 and *X*1, *X*2 and *X*3 are the values for temperature, pH and shaking speed, respectively. Analysis of variance (ANOVA) for cypermethrin degradation is presented in Table 2. Determination coefficient *R* ^2^ = 0.8863 indicated that approximately 88 % of responses were covered by the model, demonstrating that predicted values of the model were in good agreement with the experimental values. The model for cypermethrin biodegradation is highly significant (*p* < 0.0001), indicating that the established quadratic model for cypermethrin degradation by SG2 was adequate and reliable and represented actual relationship between response and variables (Fig. 3).

**Table 2:** ANOVA for the fitted quadratic model for cypermethrin biodegradation

Source	SS	*DF*	MS	F value	*P* level
Model	694.1558	9	77.12842	8.656952	0.0012
X1	5.432684	1	5.432684	0.609769	0.4530
X2	9.760639	1	9.760639	1.095541	0.3199
X3	1.402913	1	1.402913	0.157464	0.6998
X1X1	180.0615	1	180.0615	20.21024	0.0012
X2X2	548.3952	1	548.3952	61.55229	<0.0001
X3X3	3.519622	1	3.519622	0.395045	0.5437
X1X2	1.805	1	1.805	0.202595	0.6622
X1X3	0.08	1	0.08	0.008979	0.9264
X2X3	0.845	1	0.845	0.094843	0.7644
Residual	89.0942	10	8.90942		
Lack of fit	85.8942	5	17.17884	26.84194	0.0013
Pure error	3.2	5	0.64		
Cor total	783.25	19			

*R* ^2^ = 0.8863, CV = 4.07

*DF* degrees of freedom, *SS* sum of sequences, *MS* mean square

* *P* level <0.05 indicates that the model terms are significant

### FT-IR analysis of biodegraded cypermethrin

**Fig. 4:**
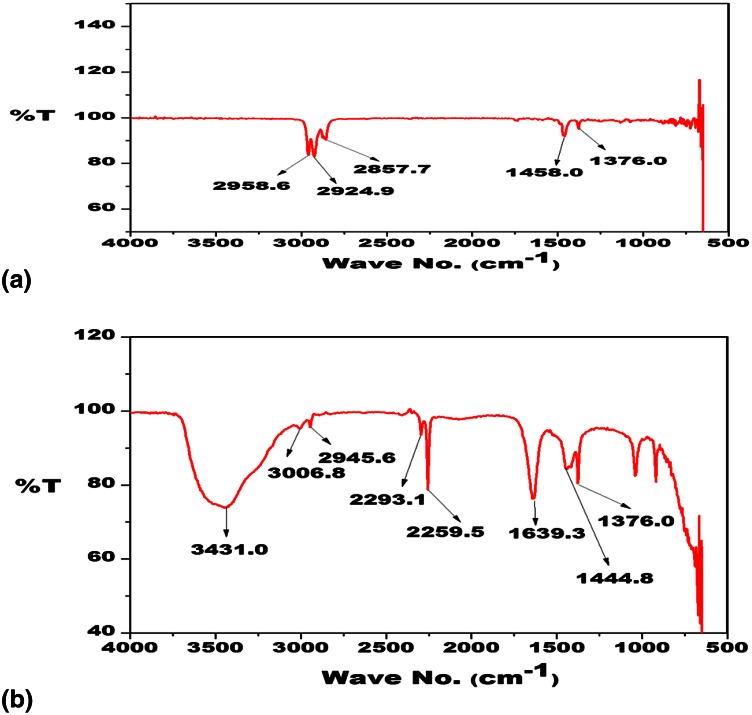
FT-IR spectra of cypermethrin. **a** Cypermethrin control. **b** Cpermethrin treated with SG2

Degradation of cypermethrin in soil slurry was maximum as compared to minimal broth (data not shown). Cypermethrin degradation in mimimal media and in soil slurry was 81.6 and 89 %, respectively, after 15 days with SG2. FTIR studies were conducted to identify the changes in bonding/streching and vibrations in biodegraded cypermethrin. Five major peaks were present at 1376, 1458, 2857.7, 2924.9, 2958.6 cm^−1^ in control. Significant changes in peak pattern of cypermethrin under bacterial treatment as compared to control were observed (Fig. 4a, b). Absorbance of the bands, associated with ether-cyanate and ester group was 1376 and 1100 cm^−1^, respectively. A decrease in cypermethrin carbonyl band (1740 cm^−1^) and a slight increase in carbonyl signals (around 1650–1700 and 1760–1780 cm^−1^) were observed due to carbonyl stretching. Stretching in C=C chloroalkenes, ring vibration of benzene, CH_2_ deformation in R–CH_2_–CN structure and (C=O)–O–stretching in cypermethrin were reported by Kaur et al. (2015) using *Fusarium* sp. In the present study, FTIR spectra of biodegraded cypermethrin showed major changes in the range of 1000–1650 and 2259–3431 cm^−1^, indicating degradation of cypermethrin. Similarly, Rosenheimer and Dubowski (2008) performed photolysis of thin films of cypermethrin using in situ FTIR monitoring. Identified photoproducts were 3-phenoxybenzaldehyde, 3- phenoxybenzoic acid, acetonitrile and cypermethrin isomers (Rosenheimer et al. 2011).

### GC–MS analysis of cypermethrin metabolites

**Fig. 5:**
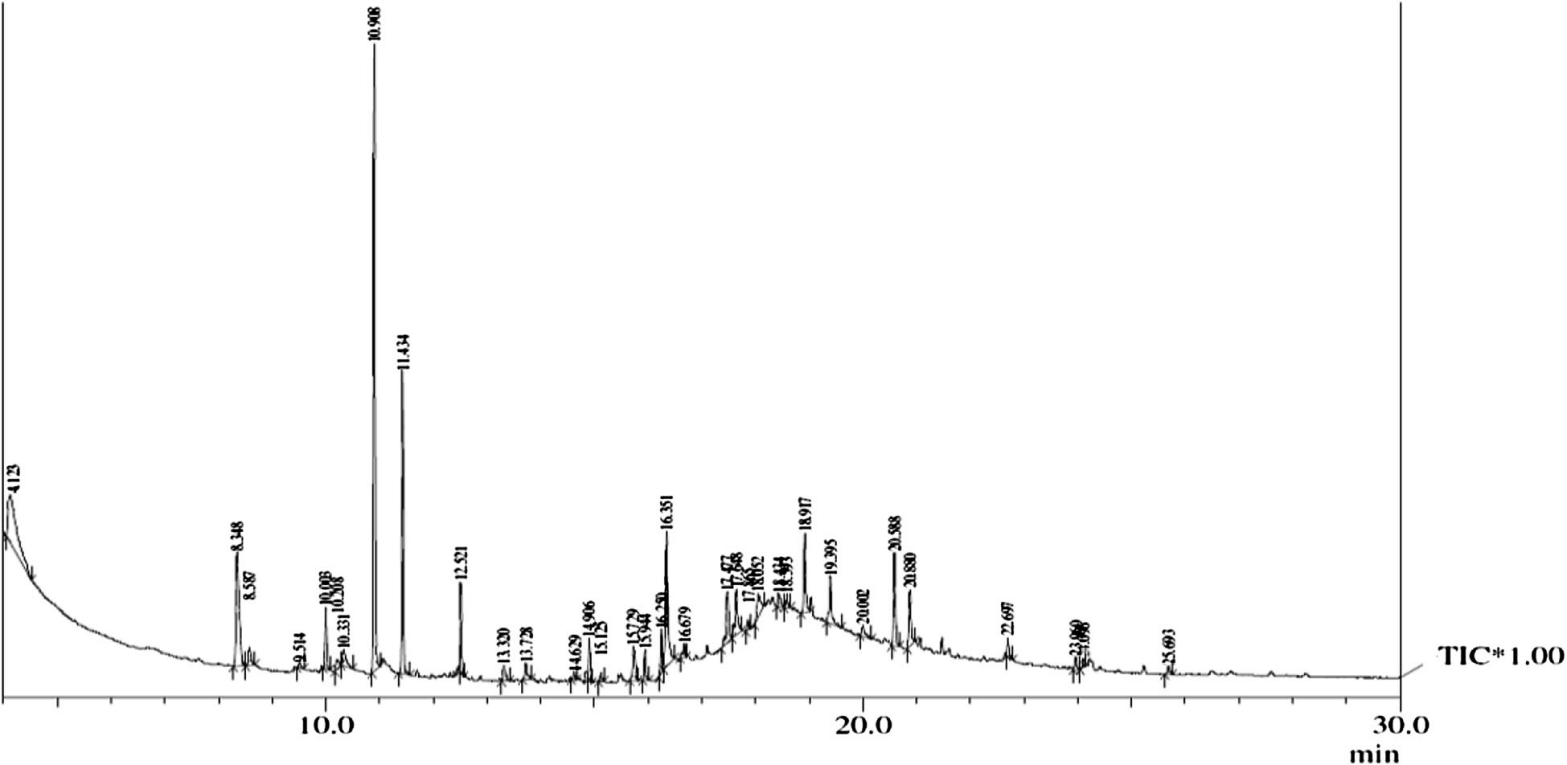
GC-MS spectra of the cypermethrin with strain SG2

Peaks of two compounds, GC1 (4.123 min) and GC2 (4.125 min), appeared first during the cypermethrin biodegradation by SG2. These compounds were identified as phenol and 4-hydroxybenzoate, respectively, based on their retention time and molecular weight with those of corresponding authentic compounds in the database. Peaks of GC3 (8.348 min) and GC4 (8.587 min) were observed and the corresponding compounds were 4-propylbenzaldehyde and phenol, M-tert-butyl as per the database. Similarly some other metabolites were also identified as they showed different retention time (Table 3). Based on the identity of the metabolites, biodegradation pathway of cypermethrin in strain SG2 was proposed and presented in Fig. 5. Cypermethrin was initially metabolized by the hydrolysis of ester linkage which yielded 3-(2, 2-dichloro ethenyl)—2,2-dimethyl-cyclopropanecarboxylate [GC15] and α-hydroxy-3-phenoxybenzeneacetonitrile [GC7]. Compound GC7 was unstable in the environment and oxidized to form 3- phenoxybenzaldehyde [GC8]. Subsequent oxidation of GC8 resulted in the formation of 4-propylbenzaldehyde [GC3]. Compound GC3 could be further transformed to 4-hydroxybenzoate [GC2], which could be converted into phenyl ester of o-phenoxy benzoic acid (GC13). Subsequently, GC13 transformed into M-tert-butyl phenol (GC4), which can further be converted to 2-tert-pentylphenol (GC5). It is possible that GC1 and GC5 transformed into small molecular weight aliphatic compounds, i.e. 1-dodecanol (GC6), isopropyl myristate (GC9) and oleic acid (GC11) and may lead to complete mineralization of cypermethrin thereafter.

**Table 3:** Identification of intermediate metabolites of cypermethrin by GC–MS

Intermediate metabolites with their code number	Retention time (min)	Molecular weight (MW)	Intermediate metabolites
GC1	4.123	94	Phenol
GC2	4.125	138	4-Hydroxybenzoate
GC3	8.348	148	4-Propylbenzaldehyde
GC4	8.587	150	Phenol, M-tert-butyl-
GC5	9.514	164	2-Tert-pentylphenol
GC6	10.908	186	1-Dodecanol
GC7	13.725	225	*α*-Hydroxy-3- phenoxy- benzeneacetonitrile
GC8	13.728	198	3-Phenoxy-benzaldehyde,
GC9	14.906	270	Isopropyl myristate
GC10	15.944	298	Hexadecanoic acid, methyl ester
GC11	18.052	282	Oleic Acid
GC12	18.917	270	Isoamyl laurate
GC13	23.700	256	Phenyl ester of o-phenoxy benzoic acid
GC14	23.958	415	Cypermethrin
GC15	24.100	236	3-(2,2-dichloroethenyl)-2,2-dimethyl cyclopropanecarboxylate

## Discussion

Numerous studies have shown the isolation and characterization of pesticide degrading bacteria from pesticide-contaminated soil, groundwater and activated sludge (Tallur et al. 2008; Chen et al. 2011b; Negi et al. 2014). *Bacillus* sp. is widely existing in nature and safely used in a variety of food products because of its nontoxicogenic and non pathogenic properties, identified by Food and Drug Administration (Gong et al. 2014; Xiao et al. 2015). *Bacillus* sp. is also reported to degrade a number of xenobiotic compounds including 4-chloro-2 nitrophenol, red M5B dye, dimethylformamide and methyl parathion (Arora 2012; Gunasekar et al. 2013). Current study provides the evidence of efficient degradation pathway of cypermethrin by *Bacillus* sp. Strain SG2. The bacteria converted cypermetrin into smaller molecular weight compounds which can further be mineralized under natural environmental conditions.

Biodegradation of cypermethrin increased with increased bacterial growth in minimal medium. Our observations are relevant with the findings of Singh et al. (2004) and Xiao et al. (2015). Strain SG2 could utilize cypermethrin as a sole carbon source and degraded it over a wide range of temperature (28–38 °C), pH (4–8) and shaking speed (90–110). The presence of longer exponential phase in the bacterial growth curve at initial higher cypermethrin concentration could be related to higher bacterial population requirement for pesticide degradation. It is proposed that higher initial bacterial counts were needed to initiate rapid degradation of the pesticide (Xiao et al. 2015). *Bacillus* sp. strain SG2 needed a longer lag phase to adapt to a new environment which induced the synthesis of degradative enzymes (Dubey and Fulekar 2013). The degradative enzymes do not show much substrate specificity, so one strain of *Bacillus* sp. can grow and degrade many hazardous substances. Strain SG2 could degrade all the pyrethroids because all the pyrethroid insecticides share a similar structure as reported by Wang et al. (2011).

The purpose to use RSM in the present study was to optimize the best culture conditions for maximum biodegradation of cypermethrin. RSM is the evaluation of relationships between the predicted values of the dependent variable and the conditions of dependent variables (Hosseini-Parvar et al. 2009). Box-Behnken design, as used commonly in industrial applications because of its economical design, requires only three levels of each factor. Chen et al. (2011b) and Xiao et al. (2015) have used RSM, based on the Box-Behnken design to optimize the degradation condition of pyrethroid insecticides at different temperature, pH and inoculum size. They described that RSM is convenient and efficient system to investigate the optimum degradation conditions for pyrethroid insecticides by different microorganisms. In our study a statistical model based on RSM was applied to optimize the cypermethrin degradation conditions by strain SG2. The model was proved to be reliable and accurate within the limit of chosen factors.

**Fig. 6:**
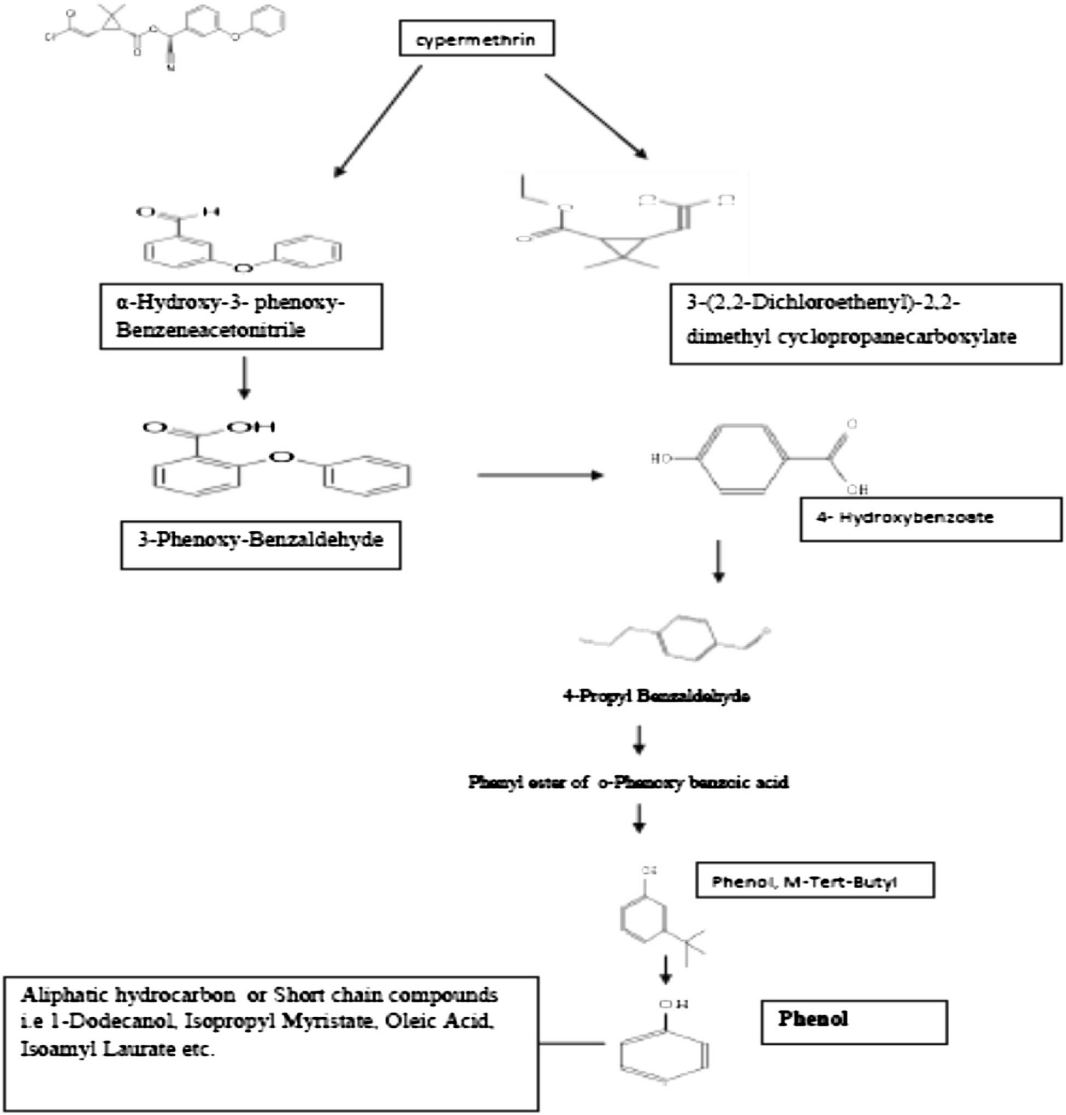
Proposed pathway of Cypermethrin degradation with SG2 strain

As a strategy to develop efficient biodegradation conditions, it is important to analyse the metabolic pathway of the test compound. Most of the pyrethroid insecticides produce more toxic intermediates in biodegradation processes (Laffin et al. 2010). Our results showed that the strain SG2 was not only efficiently able to degrade cypermethrin but also transformed the metabolites (compound GC1-GC15) into non toxic forms. Our results are in accordance to the reports of Zhang et al. (2011). It is presumed that ester hydrolysis of pyrethroid takes place by carboxylesterases. This enzyme acts as a regulatory enzyme for pyrethroid degradation and results in acid and alcohol production (Zhang et al. 2010; Xiao et al. 2015). Metabolites of biodegraded cypermethrin in soil slurry were extracted and identified by GC–MS (Table 3). Each peak was identified on the basis of its characteristic m/z fragment ions and matched with authentic compounds available in the database. Chromatogram of GC–MS for cypermethrin degradation by strain SG2 is shown in Fig. 6. Cypermethrin disappeared concomitantly with the formation of new metabolites. In the present study, cypermethrin was metabolized to several intermediate compounds which were further transformed into non- toxic and environmentally accepted forms. By arranging the metabolites into their location in the pathway we have proposed the hypothetical degradation pathway of cypermethrin using strain SG2. Maximum degradation of cypermethrin was observed in soil slurry; hence intermediate compounds of biodegraded cypermethrin were extracted from soil slurry after 15 days. Cypermethrin could be metabolised into two major compounds (α-hydroxy-3-phenoxy- benzene acetonitrile and 3-(2, 2-dichloroethenyl)-2,2-dimethyl cyclopropanecarboxylate). α-hydroxy-3- phenoxy- benzene acetonitrile is unstable and spontaneously transformed to yield 3-phenoxy benzaldehyde (Chen et al. 2011b; Lin et al. 2011; Xiao et al. 2015). 3- phenoxybenzaldehyde has antimicrobial activity, but does not affect producing culture and enhances biodegradation in soil or media (Chen et al. 2011b). 3- phenoxybenzaldehyde is transformed into 4-propylbenzaldehyde which again converts to 4-hydroxybenzoate. Afterward 4-hydroxybenzoate was metabolized by strain SG2 to form phenyl ester of o-phenoxy benzoic acid. Two intermediate metabolites (3-phenoxybenzoic acid and 3-phenoxybenzaldehyde) are the key metabolites of pyrethroids (Laffin et al. 2010). Chen et al. (2011c) reported the degradation of 3-phenoxybenzoic acid by *Stenotrophomonas* sp. strain ZS-S-01. A *Pseudomonas* sp. was also able to utilise β-cypermethrin (Halden et al. 1999). In the present study, metabolites of 3-phenoxybenzaldehyde degradation were also reported. Strain SG2 was also able to use 3-phenoxybenzaldehyde. These results demonstrate that *Bacillus* sp. strain SG2 possesses the metabolic pathway for the complete detoxification of cypermethrin, indicating that strain SG2 may be an ideal microorganism for bioremediation of the cypermethrin in contaminated soil or water system.

## Electronic supplementary material

Below is the link to the electronic supplementary material.

Supplementary material 1 (DOCX 434 kb)
