# Supplementary material for: Novel pathway of cypermethrin biodegradation in a Bacillus sp. strain SG2 isolated from cypermethrin-contaminated agriculture field
Source: 3 Biotech. 2016 Feb 4;6(1):45. doi: 10.1007/s13205-016-0372-3 (PMC4742419; doi:10.1007/s13205-016-0372-3)
Supplement: Supplementary file 1 — Supplementary material 1 (DOCX 434 kb) [file 13205_2016_372_MOESM1_ESM.docx]

**Supplementary Material:**

**Figure captions:**

**Fig.A.** The gel electrophoresis of 16S rDNA of strain SG2

2000 bp


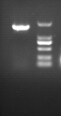


16S rDNA Marker

1417 bp

1000 bp

250 bp

100 bp

**S1. Phenol**

S2. 4- hydroxybenzoate

S3. 4-Propylbenzaldehyde

S4. Phenol, M-tert-butyl

S5. **2-Tert-Pentylphenol**

**S6. 1-Dodecanol**

**S7. α-Hydroxy-3- phenoxy- Benzeneacetonitrile**

**S8. 3-Phenoxy- benzaldehyde**

**S9. Isopropyl Myristate**

**S10.** Hexadecanoic Acid, Methyl Ester

**S11. Oleic acid**

**S12. Isoamyl Laurate**

**S13. Phenyl ester of o-Phenoxy benzoic acid**

**S14. Cypermethrin**

**S15.** 3-(2,2-Dichloroethenyl)-2,2-dimethyl cyclopropanecarboxylate
